# Specific subsets of immune cells in human decidua differ between normal pregnancy and preeclampsia - a prospective observational study

**DOI:** 10.1186/1477-7827-7-132

**Published:** 2009-11-23

**Authors:** Lorenz Rieger, Sabine Segerer, Thomas Bernar, Michaela Kapp, Monika Majic, Ann-Katrin Morr, Johannes Dietl, Ulrike Kämmerer

**Affiliations:** 1 Department of Obstetrics and Gynaecology, University of Würzburg, Josef-Schneider-Str. 4, D-97080 Würzburg, Germany

## Abstract

**Background:**

Changes in the balance of decidual leucocyte populations may lead to an unfavourable uterine microenvironment which may be associated with the development of preeclampsia (PE). In this study, we therefore investigated the leucocyte subpopulations in decidual tissues of 33 women with preeclampsia and 66 control patients.

**Methods:**

Decidua was either obtained via curettage during cesarean section or dissected from the surface of the basal plate of the placenta after spontaneous delivery. We used FACS analysis to quantify decidual leukocytes (CD45), NK cells (CD56+/CD16+ and CD56++/CD16-), antigen presenting cells (HLA-DR, DC-Sign, CD14) and T/B cells (CD8, CD4, alpha-beta-T-cell receptor, gamma-delta-T-cell receptor, CD25, CD19).

**Results:**

The number of decidual cytotoxic CD8+T-lymphocytes (P < 0.02), alpha-beta -T-cell receptor positive T cells (P < 0.03) and of CD56+/CD16+ NK cells (P < 0.03) was lower in decidua from women with PE than in decidua from control patients.

**Conclusion:**

The observed reduction of specific leucocyte subsets could create a microenvironment which is unfavourable for an appropriate placentation and could thereby be involved in the development of preeclamptic symptoms.

## Background

Preeclampsia (PE) is a major clinical problem occuring in about 5% of all pregnancies. It is often linked to high maternal and perinatal mortality and morbidity even in highly developed countries [1]. To date, iatrogenic induced delivery is still the only causal therapy.

The reasons for the development of PE are not yet completely understood. However, there is increasing evidence that immunologic incompatibilities between the mother and the developing placenta may play a major role: Multiparity and longer exposition to paternal semen prior to conception are known to decrease the risk of developing PE [2,3]. Furthermore, there is a higher risk for PE if there is short or missing preceding sperm contact, e.g. in the case of donor insemination. The same holds true for certain combinations of maternal and paternal antigens or if a women opts for a surrogate mother [1,4].

It is assumed that PE can be divided into two stages: The first stage comprises the time of placentation in the first half of pregnancy. It is characterized by a deficient invasion of extravillous trophoblasts; patients are mostly asymptomatic during this time. The second stage of PE is found in the second half of pregnancy. Due to placental oxidative stress and inflammation, mediators like soluble fms-like tyrosine kinase 1 (sFlt-1) are secreted leading to an enhanced maternal systemic inflammatory reaction which may ultimately cause the clinical signs of PE such as hypertension and proteinuria [5].

Human endometrium and pregnancy decidua harbour a wide variety of CD45^+^immunocompetent cells. Within that population, uterine natural killer (uNK) cells (CD3^-^, CD16^-^, CD56^++^) form the main subpopulation [6]. They accumulate during early pregnancy in the area of implantation and are accompanied by trophoblast invasion, which suggests that uNK cells are important for a successful gestation [7]. Nevertheless, the role these cells play during the development of PE is still unkown.

It has been suggested that uNK cells could contribute to the development of PE by inducing the lysis of trophoblast cells lacking HLA-G which could prevent an invasion of the trophoblast cells which is deep enough for a sufficient supply of the developing placenta with oxygen and nutrients [8,9]. More recently this hypothesis has been challenged by studies which showed that uNK are scarcely cytotoxic and that moreover the expression of HLA-G may be of little importance in the protection against lysis by NK cells [10-12].

Uterine NK cells are known to express killer immunoglobulin-like receptors (KIR) which may deliver inhibitory or activating signals. HLA-C - like HLA-G and HLA-E - expressed in trophoblast cells, is the dominant ligand for these receptors. It has recently been shown that some combinations of KIR on uterine NK cells and fetal HLA-C molecules may be unfavourable to trophoblast cell invasion and therby increase the risk of developing PE [13]. Uterine NK cells may also play a pivotal role in angiogenesis and artery remodelling at the feto-maternal interface by producing several cytokines like IL-8 and VEGF [14,15].

Another predominant cell type of the maternal immune system that can be found when quantifying decidual immune cells are CD3^+ ^T cells [16]. T cells account for about 10% of the decidual leukocyte population. They are able to differentiate into a Th1 and Th2 type which both secrete different patterns of cytokines. Due to the specific hormonal situation, there may be a trend towards Th2 cytokine secretion in pregnancy which seems to be reduced in PE [17,18].

The general role that decidual T cells play during normal pregnancy is still unknown. As increased levels of CD4^+ ^and CD8^+ ^T cells as well as a Th1 shift can be observed in preeclamptic pregnancies, T-cells are thought to play an important role in the development of PE by increasing the systemic inflammatory response [19,20].

CD14^+ ^mononuclear cells account for about 20% of the endometrial leukocyte population. Most of them express HLA-DR and DC-Sign (CD209), which is a marker for immature dendritic cells (DCs) [6,21,22]. In contrast to immature DCs, only a tiny amount of mature CD83^+ ^DCs are found in decidua [22,23]. A recent study has reported an upregulated expression of DC-Sign in placentas of patients with HELLP syndrome, whereas in patients with PE or IUGR no difference has been found [24].

One of the hallmarks of a successful human pregnancy is the proper invasion of the placental trophoblasts into the uterine wall. Up to now the regulation of this process has not been fully understood. However, the remarkably dense population of leukocytes found in the placental bed in direct contact to trophoblasts suggests that these cells may play a role in promoting placentation. As the decidua is seeded with a huge variety of immunocompetent cells, changes in their population might be an early event in the development of PE. In this prospective observational study, we therefore compared the decidual immune cell population of patients with PE and control.

## Methods

### Patients

The study was designed as a prospective observational study. Patients were recruited between 2003 and 2007 at the department of obstetrics and gynecology, University of Würzburg. Our investigations were approved by the local ethics committee. Inclusion criteria for the PE group were blood pressure ≥ 140/90 mmHg observed after 20 weeks of gestation and a significant proteinuria of ≥ 300 mg/24 h [25]. Inclusion criteria for the control group were blood pressure < 140/90 mmHg and the absence of proteinuria. In the PE group, 23 patients suffered from early PE (symptom manifestation before 34 weeks of gestation), 10 had late PE (symptom manifestation after 34 weeks of gestation) [26].

None of the patients included had previously known diabetes, chronic hypertension or any symptomatic infectious disease. Patients of the control group with leukocyte counts ≥ 15.000/μl and with C-reactive protein > 2,5 mg/dl in maternal serum were excluded. All placentas were examined by a pathologist. Only patients whose placenta showed no signs of infection (chorioamnionitis) were included.

The distribution pattern concerning the gestational age was roughly the same in both groups. In both groups most of the patients underwent caesarean section (91% PE group vs. 86,4% control group). Fetal growth restriction was detected in 45% of the PE group and in 12% in the control group. The main characteristics of the two groups are shown in table 1. As iatrogenic preterm delivery always needs a clear indication, we show the reasons for preterm delivery in the control group in table 2.

**Table 1:** Clinical data of the patients.

	PE	Control
Number of patients	33	66
Age	31 [27; 35]	30 [27; 34]
Gestational age	33 [29; 35]	34 [30; 37]
Birth weight	1530 [1015; 2200]	2000 [1434; 2669]
Caesarean sections (%)	90.9%	86.4%
Multiple gestation	2	26
Fetal growth restriction	45%	12%

This table shows the median age, gestational age at delivery and birth weight including 25 and 75% quartiles. The mode of delivery (rate of cesarean sections in percent) and the number of multiple gestations is also provided.

**Table 2:** Causes for preterm delivery in the control group (gestational age <34 weeks)

Indication	N
Preterm labour (single)	20
Multiple gestation	12
Fetal distress	8
IUGR	6
Fetal abnormalities	2
Placenta praevia	2
Uterine rupture	1

Reasons for preterm delivery (<= 34 weeks gestation) in the control group. The reasons are presented based on declining frequency. Note that multiple answers are possible, e.g. most patients with multiple gestation were delivered for preterm labour.

### Isolation of cells

To obtain decidual tissue, mucosal surface was either scraped off the uterine lumen via curettage during cesarean section and/or dissected from the whole surface of the basal plate of the placenta after spontaneous delivery. In preceding experiments with tissue obtained from 12 women undergoing casarean section, we had verified that both types of decidua collection revealed identical cell populations when performed on the same patient (Table 3). When decidual tissue was available from curettage, this tissue was pooled with the tissue derived from the basal plate of the corresponding placenta and processed as one sample.

**Table 3:** Comparison of the two methods of decidual cell preparation

	Decidua (uterus)			Decidua (placenta)			
Antigen	Median	1. Quart	2. Quart	Median	1. Quart	2. Quart	p
**CD45 (of all cells)**	46.1	21.2	54.7	34.0	24.7	41.3	n.s.
							
**Marker on cells in % of CD45+ cells**							
							
**CD56**	52.3	47.3	59.8	42.0	38.9	73.2	n.s.
**CD16**	25.5	9.2	33.3	17.0	14.0	26.7	n.s.
**CD56^+^/CD16^+^**	10.2	8.1	14.3	13.2	6	24.7	n.s.
**CD56^++^/CD16^-^**	40.9	36.3	52.1	33.4	24.1	38.3	n.s.
**HLA-DR**	24.2	20.6	32.1	26.4	13.1	30.7	n.s.
**DC-Sign**	8	6.8	12.1	5.9	3.0	14.7	n.s.
**CD14**	16.9	8.7	20.2	15.0	12.7	29.2	n.s.
**CD8**	27.0	23.5	33.5	28.8	23.5	34.9	n.s.
**CD4**	29.8	22.8	33.6	34.1	28.6	41.7	n.s.
**αβ-T**	37.5	33.0	45.0	40.5	32.1	46.1	n.s.
**γδ-T**	3.0	1.6	5.0	2.6	1.3	3.4	n.s.
**CD25**	5.1	2.7	6.8	3.2	1.9	6.0	n.s.
**CD19**	1.6	1.2	4	2.9	1.9	3.8	n.s.

Quart = Quartile; 1. Quart: 25%; 2. Quart: 75%. P-values were calculated by the Mann-Whitney-U-Test.

Median, quartiles and p-values of the cell populations examined by FAX analysis comparing decidua obtained from the uterine cavity via curettage and prepared from the placenta (n = 12 in both cases, gestational week: 33-39).

Decidua was obtained either via curettage during caesarean section (attachment site of the placenta plus adjacent decidua) or from the corresponding placenta via preparation of the visible decidua from the basal plate directly after delivery of the placenta. To reduce contamination with peripheral blood cells to a minimum, samples were carefully cleaned of all visible signs of peripheral blood through careful washing. Data are provided as median, quartiles and p-values. NS = difference statistically not significant.

For isolation of decidual cells, specimens were carefully dissected free of attached placental and/or myometrial tissue as well as all visible blood clots and then washed twice in PBS. The total decidual tissue (1-7 g) was then minced into fragments of approximately 1 mm^3 ^and digested for 40 minutes at 37°C under slight agitation in PBS with 200 U/ml hyaluronidase, 1 mg/ml collagenase type Ia, 0.2 mg/ml DNase I (2500 Kunitz U/mg) and 1 mg/ml bovine serum albumin/fraction V (all: Sigma-Aldrich, Deisenhofen, Germany). The cell suspension obtained was filtered through sterile stainless steel 50-μm wire mesh and washed once in PBS. The mononuclear cell population was then separated by centrifugation over a Leukocyte density gradient (density 1077, PAA, Cölebe, Germany), washed twice in PBS, resuspended at 10^6 ^cells/ml in PBS/2% human immunoglobulin (Beriglobin, Behring, Marburg, Germany) and used immediately for subsequent FACS-analysis.

### Cell labeling and FACS

Flow cytometry of isolated mononuclear decidual cells was performed with directly fluorochrome-conjugated monoclonal antibodies as listened in Table 4, and the corresponding isotype control (mixture of mouse IgG1, IgG2a, IgG2b, all: Pharmingen, Heidelberg, Germany). 5 × 10^4 ^cells per sample were stained in a volume of 50 μl with 5 μl of the appropriate antibody mixture for 30 min on ice. All Phycoerythrin (PE)-labeled antibodies were used in combination with CD45/FITC. Furthermore, CD56/PE was stained in combination with CD16/FITC to detect the classical NK cells. All incubation steps were followed by a wash in cold PBS/0.1% fetal calf serum (FCS). Stained cells were subsequently resuspended in 200 μl PBS/0.1% FCS and then analyzed in a FACScan flow cytometer (Becton-Dickinson, Heidelberg, Germany). A total of 1 × 10^4 ^cells per sample were evaluated for specific staining. Results were analyzed using the WinMDI-software (Version 2.8, ©Joseph Trotter).

**Table 4:** Monoclonal (mouse) antibodies used for FACS-analysis

Antigen	Clone	Isotype	Conjugation	Dilution	Supplier
CD4	RPA-T4	IgG1	PE	1:20	BD-Ph
CD8	RPA-T8	IgG1	PE	1:20	BD-Ph
CD14	M5E2	IgG2a	PE	1:20	BD-Ph
CD16	3G8	IgG1	PE + FITC	1:20	BD-Ph
CD19	HIB19	IgG1	PE	1:20	BD-Ph
CD25	B1.49.9	IgG2a	PE	1:10	BC
CD45	HI30	IgG1	FITC	1:20	BD-Ph
CD56	B159	IgG1	PE	1:20	BD-Ph
Dc-Sign	120507	IgG2b	PE	1:20	RD
HLA-DR	TÜ36	IgG2b	PE	1:20	BD-Ph
ab-T	BMA031	IgG2b	PE	1:10	BC
gd-T	IMMU 510	IgG1	PE	1:10	BC

Antibodies used for flow cytometric analysis of isolated mononuclear decidual cells.

BC: Beckmann Coulter, Krefeld, Germany

BD-Ph: BD Biosciences - Pharmingen, Heidelberg, Germany

RD: R&D-Systems, Heidelberg, Germany

### Statistical analysis

Cellular subtypes were quantified in relation to the total number of CD45 cells detected, and this percentage was compared by the two-tailed non-parametric Mann Whitney U test, as the Kolmogorov-Smirnoff test for normality indicated a non-normal distribution of data. P values < 0.05 were counted as significant.

## Results

**Figure 1:**
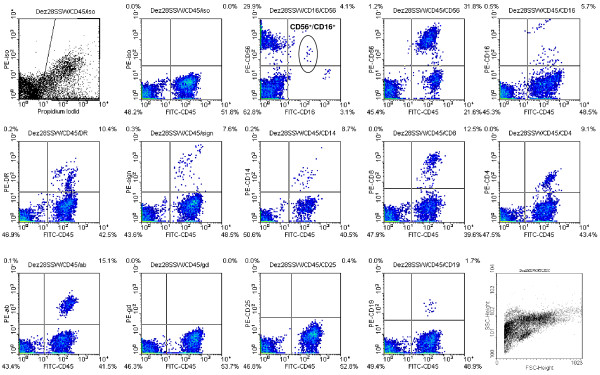
**Example of a flow cytometric analysis of a patient at 28 weeks of gestation (control group)**.

The following cell surface markers were investigated by FACS analysis: CD45, CD56, CD14, HLA-DR, DC-Sign, CD25, αβ-T-cell receptor, γδ-T- cell receptor, CD4, CD11b, CD19, CD8, CD16 and CD16^+^/CD56^+^. A typical FACS blot pattern is shown in Figure 1.

**Figure 2:**
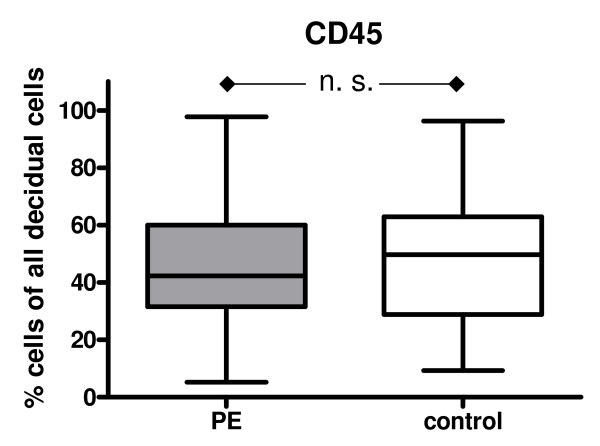
**Box and whisker plots provide the median expression of CD45 including quartiles**. The percentage is given in relation to the total cell suspension as a whole. Preeclampsia group (PE): n = 33 patients; Control group: n = 66 patients.

**Figure 3:**
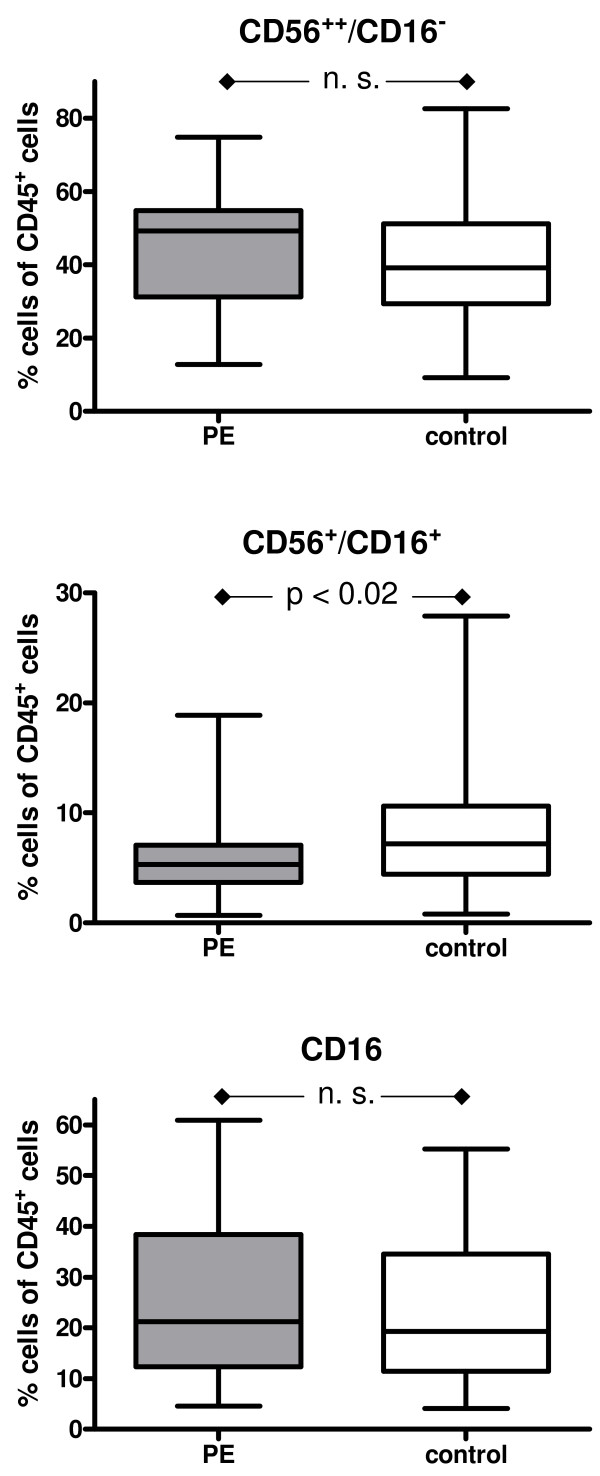
**Box and whisker plots provide the median expression of CD56/CD16, CD56/CD16 and CD 16 including quartiles**. The percentage is given in relation the CD45+ cells. Preeclampsia group (PE): n = 33 patients; Control group: n = 66 patients.

The median of the percentage of the cells detected by FACS analysis was employed. The results are summarized in Figure 2, 3, 4 &5.

**Figure 4:**
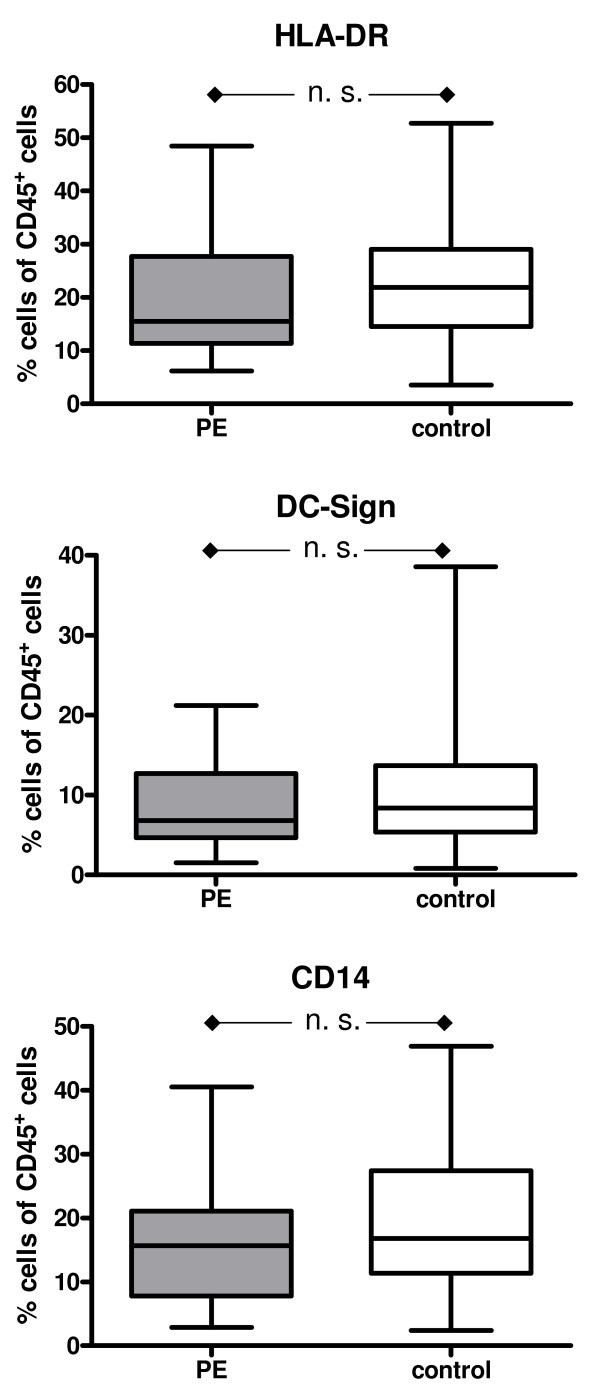
**Box and whisker plots provide the median expression of HLA-DR, DC-Sign and CD14 including quartiles**. The percentage is given in relation the CD45+ cells. Preeclampsia group (PE): n = 33 patients; Control group: n = 66 patients.

**Figure 5:**
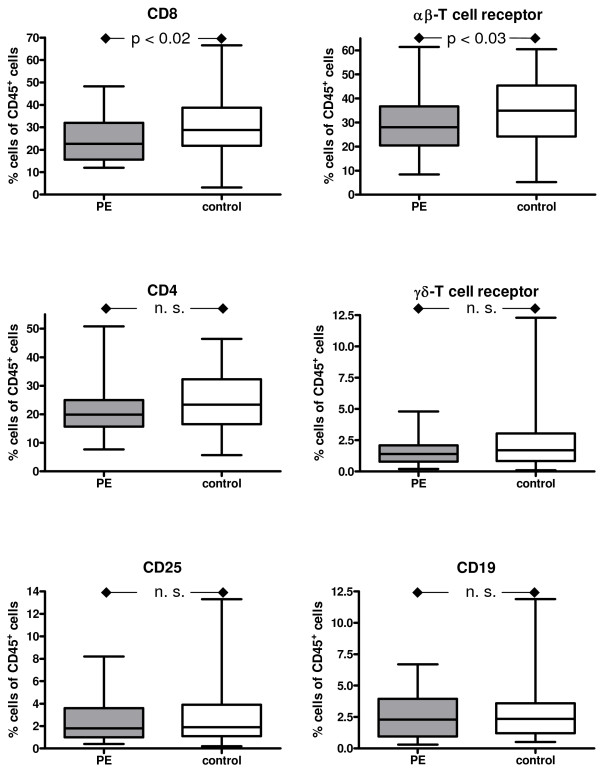
**Box and whisker plots provide the median expression of CD8, αβ-T-cell receptor, CD4, γδ-T-cell receptor, CD25, CD19 including quartiles**. The percentage is given in relation the CD45+ cells. Preeclampsia group (PE): n = 33 patients; Control group: n = 66 patients.

Although a slighthly lower number of CD 45^+ ^leukocytes was detected in the decidua of the PE group (42.3%), this was not significantly different from the result of the control group (49.8%; Figure 2). No significant difference could be observed when patients with early PE (≤ 34 weeks of gestation, n = 23) and late preeclampsia (> 10 weeks of gestation, n = 10) were compared (not shown).

### NK cells

The percentage of uNK cells (%CD56^++^/CD16^- ^in isolated CD45^+ ^cells) did not differ significantly between the two groups (44.6% control group vs 40.5% PE group), although a higher percentage was found in the PE group. However, significantly more classical CD56^+^/CD16^+ ^NK cells were detected in the control group than in the PE group (7.3% vs 5.3%, p < 0,02; Figure 3).

### Antigen presenting cells

HLA-DR^+ ^Antigen presenting cells including, DC-Sign^+ ^immature dendritic cells and monocytes expressing CD14, account for about 15% of all CD45^+ ^decidual cells and were found in lower numbers in the PE group than in the control group, although the difference did not reach statistical significance (Figure 4).

### T-cells and B cells

We found a significantly higher proportion of CD8^+ ^cytotoxic T cells in the control group than in the PE group (28,8% vs 22,6%, p = 0,02) Similarly, there were also significantly more αβ-T cells in the control group than in the PE group (35,0% vs 28,1%, p = 0,03).

In contrast to that, the small number of γδ-T cells did not differ between the groups (1,7% control group vs 1,4% PE group). Neither was the difference between the CD4^+ ^cells in the control group (23,4%) and the PE group (19,9%) significant.

The rate of CD25^+ ^cells representing either regulatory T cells or mature dendritic cells was similar in both groups (control group 1,9%, PE group 1,8%).

CD19^+ ^B cells were represented equally in both groups (2,3%; Figure 5).

## Discussion

Despite many efforts the mechanisms that cause the development of pre-eclampsia have not yet been elucidated. The great diversity of the different kinds of placentation in the animal kingdom point to the fact that in vivo models are of limited use for an understanding of the pathological processes during human placentation [27,28]. An inbuilt difficulty is that the potential immunologic malfunction occurs during early pregnancy - a period of time when the symptoms of PE are not yet apparent. That means that one can only know whether an individual person is suffering from PE when the above mentioned immunologic malfunction has already occurred. There is growing evidence that the clinical features of PE are generated either by defective placentation during early pregnancy and/or by a distinct increase in the systemic inflammatory response in the second part of pregnancy [29].

In the present study we compared the decidual immune cell populations in women suffering from PE with a control collective.

When comparing the whole cell mixture derived from the decidual samples, we found that the overall number of CD45^+ ^leukocytes was reduced - though only not significantly so in our study - in patients suffering from PE. This finding seems to confirm the observation of Williams et al. that the overall leukocyte population in the decidua of preeclamptic women is reduced [30]. Since we were interested in the relative proportion of immune cells in relation to each other, we compared the leukocyte subpopulation in relation to the total CD45 count of the respective patient by flow cytometric double staining.

We found that the percentage of CD8^+ ^and αβ-T-cell receptor positive T cells as well as that of classical CD16^+^/CD56^+ ^NK cells in relation to the number of CD45^+ ^leukocytes was significantly lower in the decidua of preeclamptic patients than in the control group. Similarly, Wiliams et al. demonstrated a significant reduction of CD8^+ ^and of CD56^+ ^lymphocytes in the decidua of preeclamptic patients by using immunohistochemistry [30]. This observation is rather surprising, since inflammation is thought to be an important component of clinically manifest PE [19,31].

However, there is evidence that an inflammatory environment is an important prerequisite for the invasion of tumour cells into healthy tissue in several cancers [32,33]. As invasive trophoblasts share many features with tumour cells, one could speculate if the diminished invasiveness of trophoblasts associated with PE might be a consequence of the lower inflammatory cell population in the placental bed.

In contrast to that, Stallmach et al., using immunohistochemistry, observed a significant increase in CD8^+ ^decidual T cells and Wilczyñski et al. found an increased percentage both of CD8^+^/CD28^+ ^T cells and CD16^+^/CD56^+ ^NK cells by FACS analysis in patients with PE [34,35]. They compared the overall amount of the cell populations and not - as we did - the relation to the total leukocyte count (CD45^+ ^cells) [35].

In our study the number of CD16^-^/CD56^+ ^uNK cells did not differ significantly in the PE and the control group. Previous studies investigating this issue by immunohistochemistry led to conflicting results: While Williams et al. and Eide et al. found a reduction, Stallmach et al. described an increase in uNK cells in cases of PE [30,34,36].

In early pregnancy, a significant increase in CD14^+^, HLA-DR^+ ^and DC-Sign^+^- antigen presenting cells can be observed [23]. Studies investigating the number of antigen presenting cells in the decidua of preeclamptic patients have shown divergent results: while two studies found an increase, three described a decrease in the number of macrophages [30,37-41].

Using immunohistochemistry, a recent study has revealed that the expression of DC-SIGN^+ ^cells is upregulated in placentas of patients with HELLP syndrome [24]. In this study, we found that the number of DC-SIGN^+ ^immature dendritic cells as well as CD14^+ ^and HLA-DR^+ ^antigen presenting cells were identical in the PE and the control group. This observation stands in contrast to a previous study which detected an increased infiltration of the decidua with both immature and mature dendritic cells in cases of PE [42].

A possible explanation for the different results could lie in the method used for the preparation of the tissue. In the above mentioned studies placental bed biopsies were obtained. Because of the invasiveness of this procedure, a contamination of the probes with peripheral blood cannot be excludued. This could result in a changed cell population. Placenta bed biopsies furthermore may contain a considerable amount of myometrial tissue which is known to contain a remarkably high number of antigenpresenting cells (manuscript in preparation). In our study, we separated the decidual tissue from the placental surface after the delivery of the placenta and/or obtained it via curettage of the uterine cavity. This method provides minimum contamination with peripheral maternal blood and therefore appears to actually reflect the real in vivo situation. Although the probes were meticulously cleaned from debris, the possibility of contaminations with peripheral blood can, however, not be completely eliminated.

Another difference between our study and the studies we mentioned above is that we used FACS analysis instead of immunohistochemistry for the quantitative analysis of the decidual cell populations. Both methods have their advantages and their shortcomings: IHC shows the spatial allocation of different cell types in the investigated tissue and may provide evidence for specific cell-cell interactions. On the other hand, FACS analysis is more accurate in terms of quantitative analysis, allows the inclusion of higher cell numbers into the calculation and has less interobserver bias.

## Conclusion

In this prospective study we compared - in a relatively large group of patients (33 patients in the PE group and 66 patients in the group without PE) - the number of various immune cells within the decidua which might be involved in the development of PE. We found that the number of CD56^++^/CD16^-^uNK cells and DC-SIGN^+ ^immature DCs does not differ significantly between the groups, whereas CD8^+ ^and αβ-T-cells as well as CD56^+^/CD16^+ ^NK cells were found less frequently in the PE group. Further investigations are needed to specify the role of these cells in physiological and pathological pregnancy.

## Competing interests

The authors declare that they have no competing interests.

## Authors' contributions

UK, JD and LR have participated in the design of the study. The experiments were carried out by MK and UK. AKM and MM provided and analysed the clinical data. TB, LR and JD helped in collecting the samples. Data analysis was performed by LR, TB and MM. LR, SS and UK drafted and wrote the manuscript. All above-mentioned authors read and approved the final manuscript.
